# Evidence for the Loss of Pneumatization and Pneumosteal Tissues in Secondarily Aquatic Archosaurs

**DOI:** 10.1093/iob/obaf039

**Published:** 2025-12-03

**Authors:** P J Byrne, N D Smith, E R Schachner, D J Bottjer, A K Huttenlocker

**Affiliations:** Department of Anatomy, New York Institute of Technology, College of Osteopathic Medicine, 101 Northern Blvd, Glen Head, NY 11545, USA; Division of Paleontology, American Museum of Natural History, 200 Central Park West, New York, NY 10024, USA; Dinosaur Institute, Natural History Museum of Los Angeles County, 900 W Exposition Blvd, Los Angeles, CA 90007, USA; Department of Physiological Sciences, College of Veterinary Medicine, University of Florida, 1333 Center Drive, Gainesville, FL 32603, USA; Department of Earth Sciences, University of Southern California, 3651 Trousdale Pkwy, Los Angeles, CA 90089, USA; Division of Integrative Anatomical Sciences, Keck School of Medicine, University of Southern California, BMT 1333, San Pablo St, CA 90033, USA

## Abstract

The evolutionary origins of the avian air sac pulmonary system are enigmatic due to the rarity of soft-tissue preservation in fossils. Here, we test whether fine anchoring fibers on the endosteal bone of bird and non-avian dinosaur vertebrae—termed “pneumosteum”—are absent in taxa lacking pneumatic openings. We studied thin sections from the caudalmost cervical and cranial dorsal vertebrae of 21 extant amniotes to infer the presence or absence of invading diverticula through vertebral foramina. We also provide a differential diagnosis of the structural features of pneumosteum. We found that the secondarily aquatic Western grebe (*Aechmophorus occidentalis*) and Magellanic penguin (*Spheniscus magellanicus*) lack external pneumaticity and pneumosteum. In addition, the small passerine bird examined (*Estrildidae* spp.) exhibits invading diverticula but no pneumosteum. This suggests that ventilatory air sacs and associated diverticula can be present despite the absence of osteological and histologic correlates and that these features are lost when transitioning to an aquatic lifestyle or in small-bodied birds. In volant pneumatized birds, diverticula and pneumosteum are associated with pneumatic foramina. This suggests that, in fossil birds, pneumatic foramina are good indicators of the presence of pulmonary diverticula. Furthermore, the loss of invading respiratory diverticula and pneumatic osteological characters in the postcranial skeleton of pursuit diving birds serves as a reminder that adaptation to specific ecologies, such as an aquatic environment, may obscure our ability to reconstruct soft tissue systems accurately in fossil taxa when relying on osteological correlates.

## Introduction

Archosaurs are a highly successful, diverse clade of reptiles that have adapted to a variety of ecosystems and environments (Fiorillo and Gangloff 2001; Brusatte et al. 2008; Padian and Woodward 2021; Navarro et al. 2022). An anatomical adaptation that may have contributed to their long-lived success is a complex, unidirectionally ventilated pulmonary system, which relies on an anatomical division between the immobilized gas-exchanging regions of the lung and highly compliant, mechanically powered air sacs (Farmer 2010; Schachner et al. 2011; Brocklehurst et al. 2020). Birds today have 9 ventilator air sacs: the cranial group, which consists of the paired cervical, unpaired interclavicular, and cranial thoracic air sacs; and the caudal group, which consists of the caudal thoracic and abdominal air sacs (Duncker 1971; Perry et al. 2019; Moore and Schachner 2025). These ventilatory sacs are flexible, bag-like extensions that branch from the immobilized, volume-constant lungs (Duncker 1971; Jones et al. 1985). These air sacs function as bellows, which pump air over the secondary bronchi and parabronchi via intercostal musculature during both phases of the respiratory cycle (Hazelhoff 1951; Brackenbury 1971; Banzett et al. 1987; Butler et al. 1988; Wang et al. 1988, 1992). The compartmentalization of these air sacs from the rest of the pulmonary system is unique among amniotes (see Moore and Schachner 2025), enabling birds to achieve highly efficient gas exchange that can facilitate behavioral adaptations suited for a variety of environments (deep ocean diving, high-altitude soaring (Parr et al. 2019), long-distance running, etc.) (Maina 2006). One of the most remarkable features of this system is the permeation of the axial and appendicular skeleton via pulmonary diverticula (air-filled epithelial protrusions) that branch off from the lungs and air sacs. This condition, known as postcranial skeletal pneumaticity (PSP), is usually associated with specific osteological features (pneumatic foramina, fossae, and laminae) formed at the site of contact on the periosteal surface (O'Connor 2004), along with a reduction of the quantity of trabecular rods in favor of increasing their average thickness and a reduction of the size of trabecular plates. Pneumatization of bone may have facilitated some of the extreme physiologies seen throughout archosaur evolution, including proposed reduction in the density and weight of bone to allow for powered flight in avian theropods and pterosaurs (e.g., Benson et al. 2012) and gigantism in sauropod dinosaurs (e.g., Sander et al. 2011). However, a few experimental studies have shown that there is no difference in mass between the skeletons of birds and mammals of the same overall mass (Prange et al. 1979; Dumont 2010; Moore and Schachner 2025) and that the question of the impact of pneumaticity on bone needs to be experimentally evaluated. While external osteological features have been used to infer the presence of an air sac-style pulmonary system in extinct non-avian dinosaurs (Wedel 2003; O’Connor and Classens 2005; Sereno et al. 2008; Benson et al. 2012), little is known regarding how intact soft tissue interacts with bone and how soft tissue resides within pneumatic recesses in extant birds. Relying on osteological characters alone to determine the presence of the air sac pulmonary system in fossil archosaurs may result in an erroneous reconstruction of the structure and anatomy of the respiratory system, as even in extant archosaurs it can be difficult to determine which soft tissues inhabit fossae and foramina along the axial column (e.g., fat deposits that reside in the lateral fossae of *Alligator* dorsal vertebrae or blood vessels that enter within nutrient foramina (O’Connor 2006)). It has only recently been appreciated that histologic correlates for pulmonary diverticula can be identified through histology (Lambertz et al. 2018). Known as “pneumosteum” or “pneumosteal tissue,” clusters of tiny scars located on the secondary trabecular and endosteal bone surfaces are hypothesized to be the attachment point of pulmonary diverticula from the air sacs and lungs. Despite pneumosteal tissue being reported in some non-avian saurischian dinosaurs and fossil birds (Lambertz et al. 2018; Aureliano et al., 2024, 2021; Brum et al. 2022; Aranciaga Rolando 2022), it is difficult to classify its morphology due to fossils undergoing taphonomy, as well as its morphological similarity to Sharpey’s Fibers. Furthermore, while Lambertz et al. (2018) groundbreaking study was able to reveal the structure of pneumosteal tissue in several bird species (i.e., *Struthio, Turdus, Buteo buteo*), it is not known if aquatic birds, which lack pneumaticity but still have air sacs, exhibit pneumosteum.

We describe the morphology of tissue located along the secondary trabecular and endosteal surface in the vertebrae of extant birds, crocodilians, mammals, and reptiles. Using extant vertebrates as a case study allows us to observe high-quality, taphonomically unaltered bone with preserved soft tissue, enabling us to ground truth the association of pneumosteal tissue with pulmonary diverticula. Furthermore, determining whether or not pneumosteum is retained or lost across secondarily aquatic transitions in diving birds and crocodylians is important when considering the evolution of the pulmonary system across terrestrial/aquatic transitions and the utility of osteological correlates.

## Materials and methods

### Sample composition

The sample is composed of a selection of birds, including members of the families: Spheniscidae, Accipitridae, Fregatidae, Turdidae, Bucerotidae, Podicipedidae, Phoenicopteridae, Psittacidae, Phasianidae, Strigidae, Estrildidae, Ardeidae, Anatidae, and Ramphastidae; crocodylians from the families: Gavialidae and Alligatoridae; mammals from the families: Tenrecidae, Heteromyidae, and Dasyuridae; and a squamate from the family Viperidae (Table 1). No IACUC was needed for these specimens because they were either collected as salvage specimens (postmortem) or are specimens from museum collections. Selected samples encompass representatives that range in both body size and life history strategies (e.g., aquatic ambush hunter, volant pursuit predator). The mammal and reptile samples included do not have the avian respiratory system, characterized by a decoupled, immobilized gas-exchanging lung and compliant, ventilatory sacs with branching epithelial tissue bodies (diverticula). Thus, we can test the validity of pneumosteal tissue as a histologic correlate, as in Lambertz et al. (2018), but with the addition of including secondarily aquatic archosaurs. All individuals are adults except *Caiman crocodilus, Gavialis gangeticus*, and *Protobothrops mangshanensis*. Juvenile individuals were included due to limitations in accessing adult samples.

**Table 1:** Specimen List and Provenience

Class	Family	Genus	Species	Specimen number	Provenience	CV	DV	SK	Sampled locality used	Reference
Aves	Spheniscidae	*Spheniscus*	*magellanicus*	KLG 451-CP	NHMLAC	X	X	NS	Entire vertebra (the neural arch pedicle is figured)	This study
	Accipitridae	*Buteo*	*buteo*	Fig. S1	IZB	X	NS	NS	Body of centrum (cervical vertebra is figured)	Lambertz et al. (2018)
	Accipitridae	*Buteo*	*jamaicensis*	T1-3 Alexander (ERS2021-001)	UF	X	NS	NS	Entire vertebra (medial to the neural arch within the pedicle is figured)	This study
	Accipitridae	*Accipiter*	*cooperii*	122826/9630; 122821/9628; 5689	NHMLAC	X	X	NS	Entire vertebra (medial to the neural arch within the pedicle is figured)	This study
	Fregatidae	*Fregata*	*magnificens*	FM01-C	NHMLAC	X	X	NS	Entire vertebra (medial to the neural arch within the pedicle and the periosteal surface of the neural arch are figured)	This study
	Turdidae	*Turdus*	*merula*	Fig. S4	IZB	X	NS	NS	Body of centrum	Lambertz et al. (2018)
	Bucerotidae	*Buceros*	*rhinozeros*	Fig. S3	IZB	NS	NS	X	Skull	Lambertz et al. (2018)
	Podicipedidae	*Aechmophorus*	*occidentalis*	122826/9630; 122834; 9632;4950	NHMLAC	X	X	NS	Entire vertebra (the neural arch pedicle is figured)	This study
	Psittacidae	ID	ID	Case1_P	SDZWA	X	NS	NS	Section of trabecular bone	This study
	Phasianidae	*Meleagris*	*californica*	LACM-E5014	LACM Rancho La Brea	NS	X	NS	Entire vertebra (the neural arch pedicle is figured)	This study
	Strigidae	*Bubo*	*virginianus*	122832/9634	NHMLAC	X	X	NS	Entire vertebra (medial to the neural arch within the pedicle is figured)	This study
	Estrildidae	ID	ID	69482-7	SDZWA	ID	ID	ID	Section of trabecular bone from a vertebra	This study
	Ardeidae	*Ardea*	*cinerea*	Fig. S1 (e-h)	IZB	X	NS	NS	Body of centrum	Lambertz et al. (2018)
	Anatidae	*Dendrocygna*	*viduata*	47281; 5350	SDZWA	ID	ID	ID	Section of trabecular bone from a vertebra	This study
	Ramphastidae	*Ramphastos*	*toco*	69119-10; 69119-11	SDZWA	X	NS	NS	Section of trabecular bone from a cervical vertebra	This study
	Phoenicopteridae	*Phoenicopterus*	*roseus*	71169-20	SDZWA	X	NS	NS	Trabeculae from the body of the centrum are figured	This study
Reptilia	Gavialidae	*Gavialis*	*gangeticus*	G.C.1.1.112322; G.C.1.3.112322; G.C.1.4.112322	NHMLAC	X	X	NS	Entire vertebra (trabeculae within the body of the centrum are figured)	This study
	Alligatoridae	*Caiman*	*crocodilus*	C.C.9.2.112322; C.C.9.3.112322; C.C.10.2.112322; C.D.1.4.112322	NHMLAC	X	X	NS	Entire vertebra (a section from within the transverse process is figured)	This study
	Viperidae	*Protobothrops*	*mangshanensis*	62509-6; 62507-7; 62508-8_1; 62508-8_2	SDZWA	X	X	NS	Entire vertebra (trabeculae from the body of the centrum are figured)	This study
Mammalia	Heteromyidae	*Perognathus*	*longimembris pacificus*	71493	SDZWA	NS	X	NS	Entire vertebra, (trabeculae from the body of the centrum are figured)	This study
	Dasyuridae	*Sarcophilus*	*harrisii*	69839-39; 69839-40	SDZWA	NS	X	NS	Trabeculae from the body of the centrum are figured	This study
	Tenrecidae	*Tenrec*	*sp.*	44226-12	SDZWA	ID	ID	ID	Trabeculae from the body of the centrum are figured	This study

The specimen list and provenience for are samples are listed as follows: Class, Family, Genus, Species, Specimen Number, Provenience, CV (Cervical Vertebra), DV (Dorsal Vertebra), SK (Skull Element), Sampled Locality, and the Reference. An X denotes which element is from that specimen. Missing information from an archived thin section is denoted as ID (incomplete data) within a box. Previously uncatalogued specimens include as much information as possible for the specimen, but lack locational context on where the section was taken from the element. This is likely due to necropsy data from zoological institutions not typically being utilized for anatomical research purposes or changes in record-keeping software during the period of time between the necropsies taking place and the analysis of the thin sections in the SDZWA collection database. The genus and species are not known for the mannikin (*Estrildidae*) or the parakeet (*Psittacidae*). In addition, it was not recorded which vertebra (cervical or dorsal) was sampled from the mannikin (*Estrildidae*), the White-Faced Whistling Duck (*Dendrocygna viduata*), and *Tenrec*. Data that was not sampled for the purposes of this study (and the other referenced studies) are denoted as NS (not sampled).

### Specimen preparation

External osteological correlates of pneumatic diverticula were recorded before destructive analysis. Samples were prepared by PJB at the Keck School of Medicine of USC and the Natural History Museum of Los Angeles County, and one sample was sent from the University of Florida (i.e., *Buteo jamaicensis*). This includes *Spheniscus magellanicus, Aechmophorus occidentalis, B. jamaicensis, Accipiter cooperii, Fregata magnificens, Bubo virginianus, Meleagris californica, G. gangeticus*, and *C. crocodilus* (see Table 1). SDZWA specimens were prepared at the Department of Disease Investigations at the San Diego Zoo Wildlife Alliance in San Diego, CA, USA. Specimens from the Steinmann Institut at Universität Bonn were studied in person and referenced from Lambertz et al. (2018), which described pneumosteum in several extant birds. In this study, the caudal-most cervical and cranial dorsal vertebrae were selected due to this region consistently being pneumatized by diverticula from the cervical air sacs or directly from the gas-exchanging lungs in extant birds. In studies done on chickens, this occurs 2 months posthatching (Hogg 1984; O’Connor 2006; Schachner et al. 2021; ERS, pers. obs.). A total of 22 sections were included in this study: 21 extant and 1 extinct (*M. californica*) (see Table 1).

A neutral EDTA solution was used to decalcify the *A. occidentalis* samples at the Keck School of Medicine. Nondecalcified bone was prepared for embedding by first immersing the bone in 10% neutral buffered formalin for 48 h, with an exchange every 24 h. The sample was then cycled through 3 stages of 70, 90, and 100% ethanol solutions for 24 h each, with a 2-min interval in a vacuum chamber prior. Samples were placed in a xylene solution for two 24-h cycles before being embedded into epoxy resin blocks. Specimens were embedded with a 2:1 ratio of AeroMarine Resin 300 and AeroMarine Hardener 21. After hardening for +8 h, pen ink was used to mark each block into sections running the length of the vertebrae. Resin blocks were then serially sectioned (cranial to caudal) using a Buehler IsoMet^TM^ Slow Speed Saw to create wafers. These wafers were mounted onto slides using 5-min Gorilla Glue before drying for up to 24 h. Subsequently, slide-mounted specimens were ground down with silicone carbide paper in a sequential fashion that decreased in density of grains to form a grinding gradient that proceeded sequentially from coarse to fine, followed by fine polishing with polishing paper to produce 100 µm-thick sections. Decalcified bone was first embedded in paraffin wax, and a microtome was used to cut wafers from the block to a thickness of 4–5 µm. Ground thin sections were visualized using a light microscope and scanned using Leica’s LAS X Suite software. Sections were imaged at varying magnifications (i.e., 5×, 10×, 20×) to visualize the gross microstructure of the bone and the secondary trabecular and endosteal surfaces in fine detail. A circularly polarized light (CPL) with a lambda filter was used to look for changes in collagen fiber orientation, which is a commonly used and highly documented technique for investigating the microanatomy of bone tissue (e.g., Bromage et al. 2003; de Buffrénil et al. 2021). When applying CPL to standard ground sections, a calibrated color representation of collagen fiber orientation will be displayed, in which different colors of collagen fiber units reflect different orientations/variations from anisotropy (de Buffrénil et al. 2021). In the past 10 years, this has been used to search for pneumosteal tissues in fossil avemetatarsalians (Lambertz et al. 2018; Aureliano et al. 2021, 2024). While most samples were sectioned in the transverse plane, several SDZWA samples were sectioned in the sagittal plane (i.e., *Protobothrops, Perognathus*, and *Sarcophilus*). This was performed as part of the necropsy process performed at the veterinary hospital of the zoo to identify and investigate instances of intervertebral disk disease (IVDD).

### Bone histology and tissue type classification

**Fig. 1:**
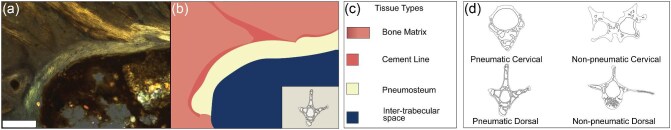
Example of a tissue map of the extinct Californian turkey (*Melagris californica*) (**a**) is a thin section under polarized light with a lambda filter. Following this is the tissue map visualizing tissue organization (**b**). Tissue types are listed next (**c**), followed by the classification of vertebral types sectioned (**d**). The scale bar is 100 μm.

Bone tissue type is characterized by a unique arrangement of collagen fibers, the orientation of ellipsoid osteocytes regarding the surrounding fibers, and whether a distinct cement line constitutes the base of pneumosteal tissues. To categorize the secondary trabecular and endosteal surface, we used tissue type classifications described in  Warshaw et al. (2017) and Warshaw (2007), which are modified from de Ricqlès and colleagues (de Ricqlès 2021). These tissue maps allow for a qualitative visualization of pneumosteum compared to other hard tissue bone matrices, soft tissue types (i.e., diverticula, marrow), and intertrabecular space (Fig. 1). While the endosteal surface of both the cortical and trabecular bone was examined, we focused on describing the morphology of the secondary endosteal surface of trabecular bone due to the availability of high-quality samples. Several specimens from the San Diego Zoo Wildlife Alliance did not have high-quality slides that contained large regions of cortical bone (i.e., *Turdus, Estrildidae, Dendrocygna, Ramphastos*, and *Sarcophilus*), as these slides were initially created during postmortem necropsies, in which the veterinary team evaluates portions of the vertebral column for trabecular pathologies.

Pneumosteum has been described in detail for saurischian dinosaurs (Lambertz et al. 2018; Aureliano et al. 2020, 2021, 2024). However, the resulting postmortem taphonomy and a lack of preserved soft tissue obscure the soft tissue/bone interface. It is therefore necessary to reassess the presence and morphology of pneumosteum in extant archosaurs to understand if this is a reliable correlate for pulmonary diverticula and to understand how taphonomic preservational biases reorganize bone microtexture (Aureliano et al. 2020).

**Fig. 2:**
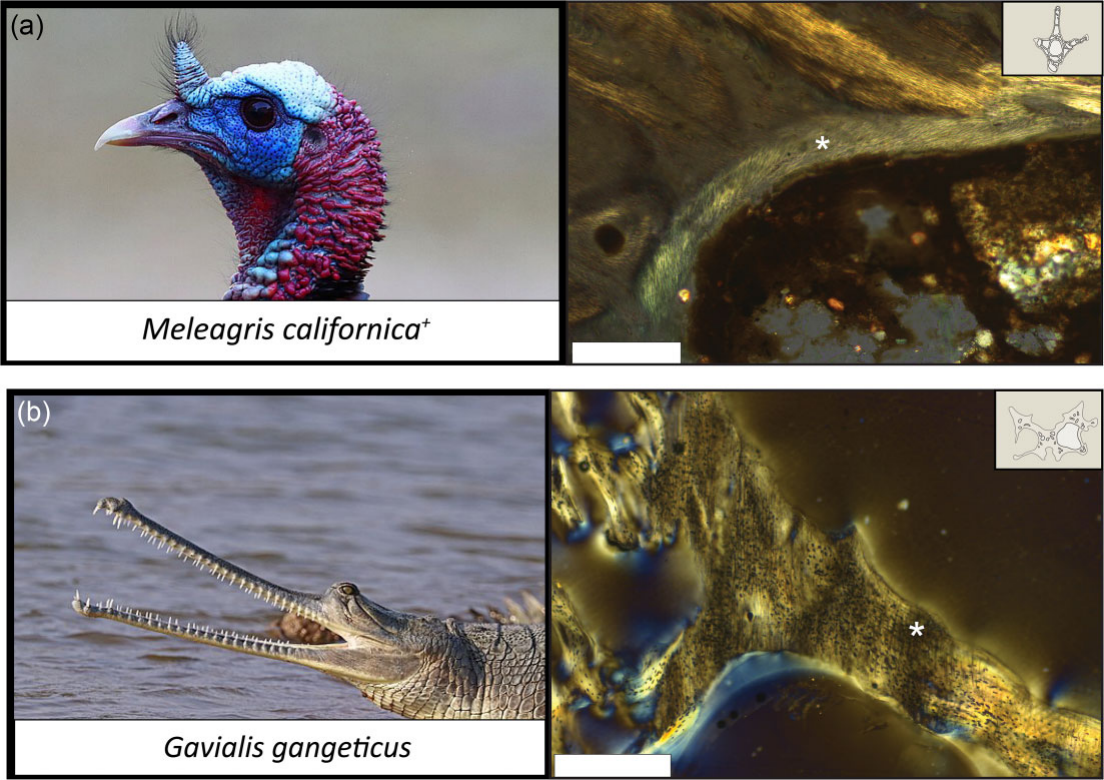
A side-by-side comparison of pneumosteal tissue in the extinct Californian turkey (*Meleagris californica*) (**a**) and a gharial (*Gavialis gangeticus*) (**b**). Pneumosteal tissue and Sharpey’s fibers are marked by an asterisk (a and b, respectively). Scale bars represent 100 μm (*Melagris*) and 300 μm (*Gavialis*). Images used from Wikimedia Commons.

Pneumosteum in fossil samples has been differentiated from Sharpey’s fibers and lamellar bone fibers by requiring a minimum magnification of 40×, having a fiber length of shorter than 60 µm, exhibiting a low optical relief, having exclusively undulose extinction, and having an asbestiform (chaotic) texture/pattern (Fig. 2) (Aureliano et al. 2024). Pneumosteum differs from other hard tissue types by exhibiting parallel fibers inclined at a 30–45° angle; it lines the edge of secondary trabecular and endosteal bone and is absent from primary bone (Table 2).

**Table 2:** Diagnosis of Pneumosteal Tissue

Feature	Description
1	Minimum magnification of 40× needed to view pneumosteum (Aureliano et al. 2024)
2	Morph A pneumosteal tissue fibers visible are typically shorter than 60 µm in length (Aureliano et al. 2024). Morph B fibers layer/fold onto each other, and can be greater than 100 µm in length (Figs. 3g and 5) (this study)
3	Low optical relief (Aureliano et al. 2024)
4	Undulose extinction (Aureliano et al. 2024)
5	Two pneumosteal tissue morphs: 1. (A) Asbestiform (chaotic) tissue texture/pattern (Aureliano et al. 2024). 2. (B) Smooth, finely laminated pneumosteal tissue morph (this study)
6	A 30–45° inclination of the fibers against the endosteal surface (Lambertz et al. 2018). This is only true for Morph A (this study)
7	Ocassional presence of pneumosteum along the periosteal surface of the cortical bone (Fig. 6) (this study)
8	Pneumosteal tissue fibers are much thinner than Sharpey’s fibers (Lambertz et al. 2018)
9	Present on secondary trabecular and secondary endosteal bone (Lambertz et al. 2018)
10	Not present in pneumatized mammalian or crocodylian skull bones (Lambertz et al. 2018)
11	Osteocytes in pneumosteal tissue are slanted at a 30–45° angle (mirroring pneumosteal tissue fibers) (this study)
12	The base of pneumosteal tissue is separated from the underlying collagen fiber matrix via a thin cement line (this study)

A comprehensive diagnosis of pneumosteal tissue. Included are diagnoses from other studies describing pneumosteal tissue (i.e., Lambertz et al. 2018; Aureliano et al. 2024).

## Results

### Patterns of pneumosteal tissue orientation

**Fig. 3:**
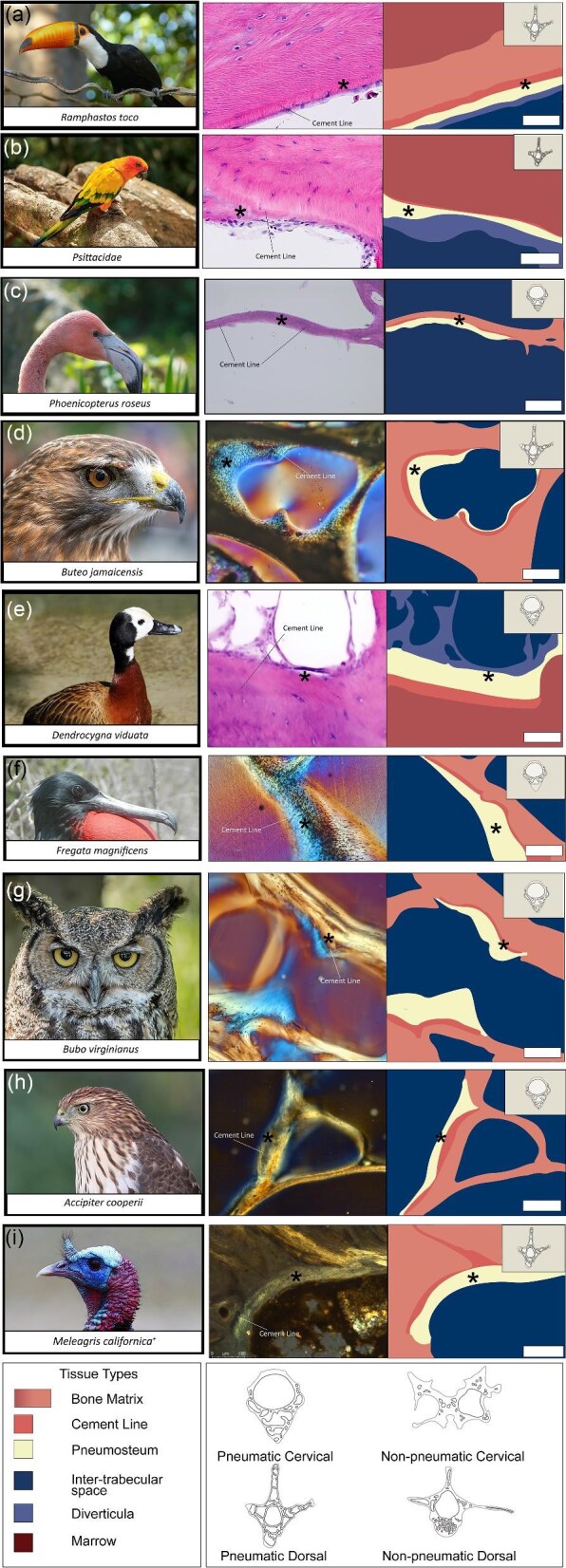
Thin sections and tissue maps of volant birds (**a**–**i**). Scale bars are as follows: a (50 μm), b (50 μm), c (100 μm), d (250 μm), e (50 μm), f (100 μm), g (200 μm), h (100 μm), and i (100 μm). Images used from Wikimedia Commons and from the San Diego Zoo Wildlife Alliance.

**Fig. 4:**
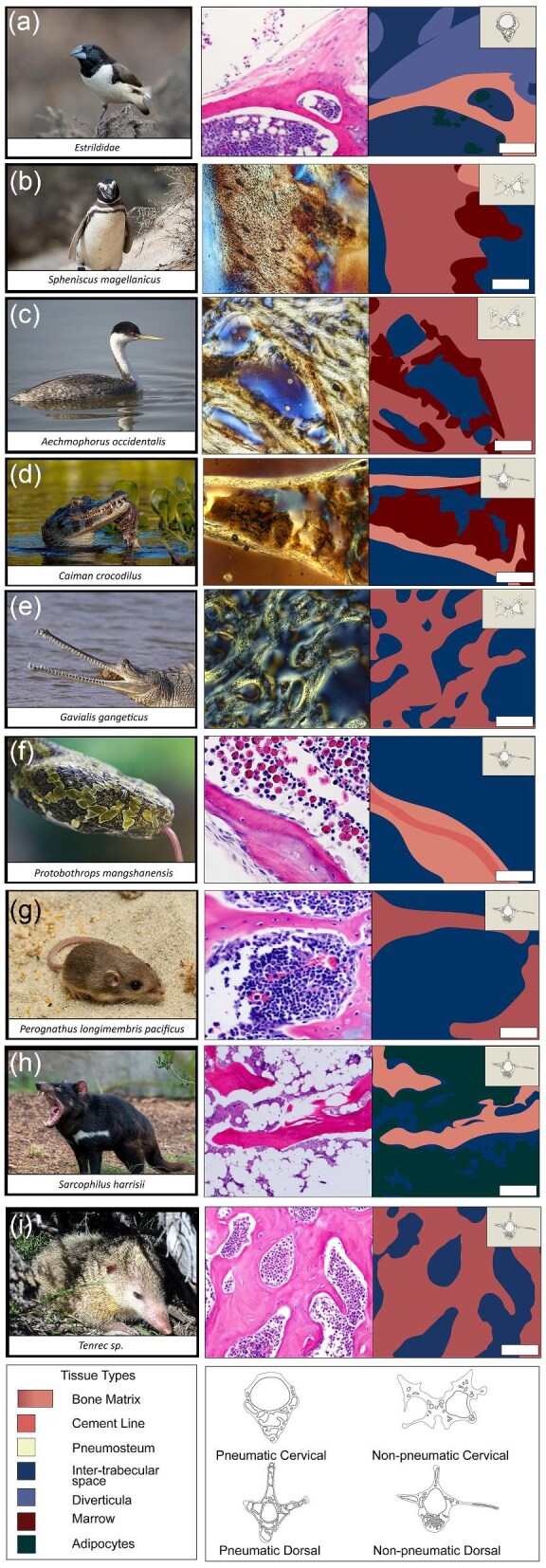
Thin sections and tissue maps of a passerine mannikin bird (**a**), semiaquatic birds (**b, c**), non-avian reptiles (**d**–**f**), and mammals (**g**–**i**). Scale bars are as follows: a (100 μm), b (166 μm), c (100 μm), d (150 μm), e (100 μm), f (50 μm), g (50 μm), h (100 μm), and i (150 μm). Images used from Wikimedia Commons and from the San Diego Zoo Wildlife Alliance.

**Fig. 5:**
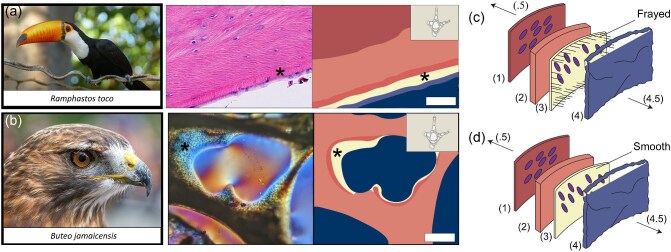
Morph A versus Morph B pneumosteal tissue. (**a**) Morph A (*Ramphastos toco*) is visualized without polarized light and is followed by a corresponding tissue map. (**b**) Morph B (*Buteo jamaicensis*) is visualized under CPL, followed by its corresponding tissue map. (**c**) and (**d**) are graphic interpretations of the morphology of pneumosteal tissue showing both frayed, chaotically organized Morph A and smooth, laminated Morph B. (0.5) represents further within the bone, while (4.5) represents toward the secondary trabecular/endosteal surface. (1) is bone matrix, (2) is a cement line, (3) is pneumosteum/pneumosteal tissue, and (4) is pulmonary diverticula. The black lines represent the alignment and texture of the fibers making up pneumosteum, and the purple ovals are osteocytes. The “Frayed” marker in the pneumosteal tissue layer (c) represents the “zipper-like” typical frayed alignment and texture of the fibers making up the pneumosteum. The “Smooth” marker in the pneumosteal tissue layer (c). (d) represents the smooth, finely laminated clusters of pneumosteal tissue fibers. Tissue layers were acquired from Servier Medical Art. Scale bars are (a) (50 μm) and (b) (250 μm). Images used from Wikimedia Commons.

**Fig. 6:**
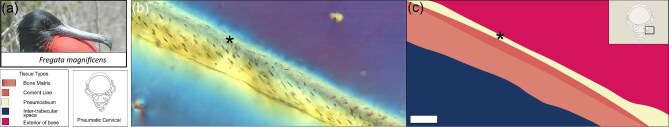
Thin section (**a**) and tissue map (**b**) depicting the periosteal surface of the cortical bone located directly below the neural arch region in *Fregata magnificens*. Scale bar is 50 μm. Image used from Wikimedia Commons.

Visualization of the secondary trabecular and endosteal surfaces results in the identification of pneumosteum in all but two of the extant volant birds that exhibit large pneumatic cavities within the cervical and dorsal vertebrae. Specifically, pneumosteum is located on the endosteal surfaces of trabecular bone bordering pneumatic cavities medial to the neural arch laminae within the pedicle and the body of the centrum of the caudal cervical and cranial dorsal vertebrae sampled—corroborating previous studies that suggest pneumosteum is a valid histologic correlate of pulmonary diverticula (Fig. 3) (Lambertz et al. 2018, Aureliano et al. 2020, 2021, 2024). All samples with pneumosteum exhibit a similar pattern of tissue deposition: a cement line that separates lamellar, woven, and parallel-fibered bone matrices from pneumosteum, which is connected to pulmonary diverticula in our decalcified samples (Figs. 2 and 3). Previously unnoticed due to taphonomic preservational bias, we note that pneumosteum exhibits 2 morphologies of collagen fiber orientation, here classified as “Morph A” and “Morph B” (Fig. 5). Morph A pneumosteum is defined as the previously described asbestiform (chaotic) texture/pattern in which pneumosteum appears to have a “zipper-like” frayed fiber structure where pulmonary diverticula attach (Fig. 5a and c). Morph B lacks this “zipper-like” fraying and exhibits smooth, finely laminated clusters of pneumosteal tissue fibers (Fig. 5b). These finely laminated clusters form a layer close to twice as thick as the layers made of the asbestiform fiber morphology. Pneumosteal tissue in the form of Morph B can be differentiated from parallel-fibered matrix by exhibiting a separation in bone matrix via a cement line—resulting in a disconformity of unaligned osteoblasts with unordered collagen fiber networks. When compared to the morphology of lamellar matrix, pneumosteal tissue from Morph B is similar in that, at low resolution, a series of stacked/folded strata are visible. However, these strata are not well defined or differentiated within the pneumosteum. Rather, these folded collagen fiber networks resemble a fan that spreads into the pneumatized cavity, often resulting in distinct peaks of clustered collagen fiber bundles. In summary, the most definite way to identify Morph B pneumosteum is by locating a defined cement line, followed by a sudden shift in the orientation of the surrounding osteocytes, exhibiting undulose extinction under CPL (Fig. 5b and d). Morph B pneumosteum is found in *B. buteo, B. jamaicensis, F. magnificens*, and *B. virginianus*. A pneumosteum is found in *A. cooperi, M. californica, Ardea cinerea, Phoenicopterus rosens*., *Ramphastos toco, Psittacidae* spp., and *Dendrocygna viduata*. However, in the instance of *F. magnificens*, both Morph A and Morph B are present in the same sample, with Morph A pneumosteum being present on the secondary endosteal surface of trabecular bone bordering internal pneumatic chambers (Fig. 3f) and Morph B appearing on the periosteal surface of the cortical bone below the neural arch region (Fig. 6). All members of Mammalia (*Perognathus longimembris pacificus, Sarcophilus harrisii, Tenrec* sp.), both crocodilians (*G. gangeticus* and *C. crocodilus*), the squamate (*P. mangshanensis*), the 2 semi-aquatic birds examined (*S. magellanicus, A. occidentalis*), and the 2 volant passerine birds (*Estrildidae* spp., this study; *Turdus merula*, described in Lambertz et al. 2018; Fig. S4) do not exhibit pneumosteum within the sampled elements (Fig. 4). This includes both the secondary endosteal and trabecular bone within the vertebrae as well as externally on the periosteal surface of the cortical bone. Intertrabecular spaces in the vertebrae of the mammal, crocodilian, squamate, and semi-aquatic bird samples are filled with marrow and blood. Notably, *Spheniscus, Aechmophorus, Estrildidae* spp., and *Turdus* have a fully formed, extensive air sac pulmonary system but do not exhibit pneumosteum. Furthermore, noninvasive diverticula from the respiratory system of *Spheniscus* and *Aechmophorus* do not create pneumatic foramina, laterally excavated fossae, or extended laminae, and do not pneumatize the neural arch region or centrum of the sampled elements.

### External pneumatic characters and association with pneumosteum

Volant avians that exhibited unambiguous external pneumatic characters (i.e., pneumatic foramina) on the surface of the cortical bone show evidence of pneumosteum on the endosteal surfaces of trabecular bone bordering pneumatic cavities medial to the neural arch laminae within the pedicle and within the body of the centrum of the caudalmost cervical and cranial dorsal vertebrae sampled. The only osteological character shared among all specimens exhibiting pneumosteum was the presence of pneumatic foramina. This is consistent with prior studies (e.g., O’Connor 2006) suggesting pneumatic foramina, when associated with direct connections leading into subdivided pneumatized chambers, are an unambiguous osteological correlate of the presence of pulmonary diverticula invading bone throughout the pneumatized cavity. For nondecalcified specimens, it was impossible to tell which diverticulum pneumatized the bone due to a lack of soft tissue preservation. Furthermore, specific diverticula were not recorded before thin sectioning the decalcified samples from the SDZWA as a part of the routine necropsy process undertaken by the veterinary pathology team.

## Discussion

### Differentiation in pneumosteal tissue types

Both Morph A and Morph B pneumosteal tissue types can be differentiated from Sharpey’s fibers associated with tendinous insertions of muscles by being much finer and thinner in structure (shorter than 150 µm versus longer than 200 µm; Figs. 2 and 5). However, it is unclear why two distinct morphotypes are exhibited. Neither morph was restricted to a specific vertebral element (i.e., cervical or dorsal vertebrae) or location within the vertebrae (i.e., neural arch region or body of the centrum). Rather, Morphs A and B were distributed throughout both cervical and dorsal vertebral elements across the pneumatic birds examined. Future work is needed to understand why one morph or the other appears in some taxa and not others and to determine if this variation is influenced by body size, ontogeny, vertebral morphology within species, or ecology. Furthermore, it is necessary to probe whether these morphs represent variations in the transverse or sagittal angulation of the cut wafer from the block, or if the ground section exhibits a minor variation in thickness, which could bias the morphology of the pneumosteum. In addition, the presence of both of these morphs in a sample, such as in *Fregata* (Fig. 6), warrants further inquiry.

In agreement with Aureliano et al. (2024), we find that a minimum magnification of 40× is required to view Morph A (asbestiform) pneumosteum. The outline of the Morph B pneumosteal tissue matrix can be visualized at a lower resolution, as the distinctive fan-like patterning of the longer collagen fiber clusters can be distinguished easily as tapering “peaks,” but it is necessary to magnify to at least 40× to confidently distinguish Morph B pneumosteal tissue from lamellar bone matrix. Furthermore, identification of slanted osteocytes associated with shifts in collagen fiber orientation directly preceding a cement line can only be clearly visualized at higher resolutions (e.g., 100×). While we agree that pneumosteal tissue appears to generally have low optical relief in both extant and fossil samples (Aureliano et al. 2024), it is important to recognize that the extremely low optical relief seen in some of the fossil samples may partially be a result of taphonomic processes influencing the preservation and subsequent visualization of clear pneumosteal tissue morphology.

In agreement with Lambertz et al. (2018), we verify that pneumosteal tissue fibers can be distinguished from Sharpey’s fibers based on length and thickness, with Morph A and B pneumosteal tissue fibers being between 60 and 100 µm in length, while Sharpey’s fibers are usually longer than 200 µm in length. In both morphs, pneumosteal tissue fibers are densely packed and clustered closely together, while Sharpey’s fibers may exhibit spacing between fibers (e.g., 10 µm of spacing, Fig. 2b). As in Lambertz et al. (2018), we also find that Morph A pneumosteal tissue fibers are inclined at a 30–45° angle against the endosteal surface (Fig. 3).

### Secondarily aquatic adaptations and presence of pneumosteum

Secondarily aquatic amniotes offer profound insight into macroevolutionary mechanics because of the requirements needed to adapt to persist under new physiological constraints of an aqueous medium (Bautista et al. 2021; Motani and Vermeij 2021). It is already well known that body mass and foraging ecology are determinative factors in patterns of pneumaticity in waterbirds due to pursuit diving for food (Smith 2012). Both *Spheniscus* and *Aechmophorus* lack PSP and pneumosteum, but both still possess an air sac respiratory system. Furthermore, the intertrabecular space in our thin sections is occupied by bone marrow (Fig. 4). This suggests that secondarily adapting to an aquatic environment inhibits the invasion of bone via pulmonary diverticula, nor do diverticula leave external markers on the periosteal surface of the exterior cortical bone in many, but not all, secondarily aquatic pursuit diving birds. This strengthens the hypothesis associating pneumatic foramina as an unambiguous osteological correlate to pulmonary diverticula and is a reliable starting point when attempting to reconstruct pulmonary diverticula/bone relationships in fossil archosaurs. However, this does not imply extinct archosaurs with pneumatic foramina must have had an air sac pulmonary system. Medially directed parabronchial protuberances branching out from the lungs, in addition to air sacs, extend tissue that invades the axial skeleton (Schachner et al. 2021).

### Limitations

We provided evidence for the absence of pneumosteal tissues in several secondarily aquatic archosaurs and within a small passerine manakin, which invites fresh perspectives on the fossil record. Yet, we acknowledge that, when assessing vertebral microanatomy, precise sampling location is crucial, particularly for detecting the interplay between bone and minute soft-tissue scars (e.g., pneumosteum and Sharpey’s fibers). Our results must be considered preliminary, since examining several locations within one sample in a vertebra (either cervical or dorsal alone), from each taxon, could be insufficient in certain scenarios: pneumaticity varies not only along the axial column but also within individual vertebrae, notably between zones of neural arches and centra (Moore 2021; Aureliano et al. 2023, 2024), as well as intraspecifically within the axial skeleton regionally and bilaterally (Lawson et al. 2025). This nuance is often overlooked. For instance, the caudalmost dorsal vertebrae of the extinct neotheropods *Allosaurus* and *Majungasaurus* possess apneumatic centra yet exhibit pneumatized neural arches (Aureliano et al. 2024). Thus, comparing apneumatic centrum trabeculae from an *Allosaurus* with pneumatized neural-arch trabeculae of a *Majungasaurus* cervical could misleadingly suggest that *Allosaurus* lacks pneumaticity, while the latter alone would be considered pneumatic. Understandably, this becomes problematic when attempting to reconstruct soft tissue structures in fossil archosaurs when preserved soft tissue is lacking. Therefore, additional rigorous testing to determine the validity of osteological correlates using extant archosaurs is required, where the association of soft tissue with bone can be easily ground-truthed. Moreover, the distinction between cortical and trabecular (compactness of spongiosa) tissues demands attention: cortical bone, even when in direct contact with external pneumatic fossae, showed no pneumosteum in the titanosaur *Arrudatitan* (Aureliano et al. 2024), but pneumosteum appears on the periosteal surface of the cortical bone below the neural arch of *F. magnificens* (Fig. 6). Despite no pneumosteal tissue appearing on the secondary endosteal, trabecular, or periosteal surface of bone in the apneumatic birds sampled in this study, whether or not a potentially pneumatizing diverticulum adjacent to a bone could leave signatures on the external cortical surface without bringing about invasive pneumatization is a question that needs further consideration in the future. Complex, apneumatic trabecular architectures have also been documented near the cotyles of various extinct non-ornithodiran archosauromorphs and archosaurs (O’Connor 2006; Butler et al. 2012). Crucially, such apneumatic trabeculae may coexist with unambiguous PSP (sensu O’Connor 2006). Furthermore, multiple types of unambiguous pneumatic features can be present in the same organism, and this may differ based on the pneumatized bone’s positioning along the axial and appendicular skeletons, its external morphology, and where the diverticulum is attaching (i.e., Morph A on the periosteal surface and Morph B on the endosteal surface in the same vertebral sample of *Fregata*; Fig. 6). Lastly, necropsy samples from zoological institutions are not typically used for large-scale comparative anatomical studies that place an emphasis on evolutionary biology. This is because the necropsy process is a screening mechanism for assessing cause of death, and specific research-focused data collection is typically not the primary objective. This, along with changes in record-keeping software during the period of time between the necropsies taking place and the analysis of the thin sections in the collection’s database, resulted in several instances of incomplete data, in which several samples did not have genus or species-level classification nor a specifically identified locality where the sample was taken within the vertebral column (see Table 1). Future work should therefore integrate more precise within-element sampling, distinguishing between cortical and trabecular tissue and zones within vertebrae (e.g., neural spine, transverse processes, and articulations in the centrum), and consider complex trabecular geometries to advance testing the histological correlates of pneumatic diverticula.

## Conclusion

Following Lambertz et al. (2018), we investigated the morphology of pneumosteum within extant birds with a special emphasis on visualizing whether pneumosteal tissues are present in semi-aquatic birds. Sharpey’s fibers can be distinguished from pneumosteum in that they do not exhibit fibers or osteocytes inclined at 30–45°, they have a larger size than that of pneumosteal tissue (larger than 60 μm), and they do not have an underlying cement line layer. Within the sampled taxa exhibiting pneumosteum, external pneumatic foramina were also found to be present. Pneumosteum was most commonly found within the neural arch region and the centrum within volant birds. This suggests that pneumatic foramina are indeed unambiguous indicators of the pneumatization of bone and further reinforces the hypothesis that pneumatic foramina are associated with pneumosteum, as they act as the passage through which pulmonary diverticula or lung diverticula invade bone.

Conversely, none of the nonpneumatic semi-aquatic archosaurs sampled (i.e., *C. crocodilus, G. gangeticus, A. occidentalis, S. magellanicus*) exhibited pneumosteum. This includes both *Spheniscus* and *Aechmophorus*, which exhibit neither pneumaticity nor pneumosteum but still have the avian-style respiratory system. The fact that pneumaticity and pneumosteum may be absent, but the avian-style respiratory system is still present, warrants caution when interpreting the presence/absence of the avian-style respiratory system within extinct non-avian dinosaurs when relying only on the presence or absence of osteological features. In addition, passerine birds like *Turdus merula* (Lambertz et al. 2018; Fig. S4) and *Estrildidae* spp. (Fig. 4a) do not exhibit pneumosteal tissue. We agree with Lambertz et al. (2018) in recognizing that body size may play a role in the presence of pneumosteal tissue bundles.

Future work on pneumosteal tissue will be focused on investigating why variations in pneumosteal tissue morphs are present and whether the presence or absence of pneumosteal tissue can differ based on taxonomy, body size, ecology, or the type of pneumatizing diverticulum. Further validating and understanding why pneumosteal tissue is present will not only allow us to better understand this new tissue type but also aid in reconstructing the pulmonary anatomy of extinct birds and non-avian dinosaurs.

## Supplementary Material

obaf039_Supplemental_Files
